# An anatomy ontology to represent biological knowledge in *Dictyostelium discoideum*

**DOI:** 10.1186/1471-2164-9-130

**Published:** 2008-03-18

**Authors:** Pascale Gaudet, Jeffery G Williams, Petra Fey, Rex L Chisholm

**Affiliations:** 1 dictyBase, Northwestern University, Center for Genetic Medicine, 676 N. St. Clair, Chicago, IL, 60611, USA; 2 School of Life Sciences, University of Dundee, Dow Street, Dundee, DD1 5EH, UK

## Abstract

**Background:**

*Dictyostelium discoideum *is a model system for studying many important physiological processes including chemotaxis, phagocytosis, and signal transduction. The recent sequencing of the genome has revealed the presence of over 12,500 protein-coding genes. The model organism database dictyBase hosts the genome sequence as well as a large amount of manually curated information.

**Results:**

We present here an anatomy ontology for *Dictyostelium *based upon the life cycle of the organism.

**Conclusion:**

Anatomy ontologies are necessary to annotate species-specific events such as phenotypes, and the *Dictyostelium *anatomy ontology provides an essential tool for curation of the *Dictyostelium *genome.

## Background

*Dictyostelium discoideum *is a facultative multicellular organism with a life cycle consisting of two mutually exclusive states, vegetative growth and development. During the vegetative cycle, *Dictyostelium *amoebae live as single cells that feed on bacteria and divide by binary fission. When environmental conditions are harsh, single-celled *Dictyostelium *amoebae enter a simple developmental pathway leading to the formation of a multicellular fruiting body composed of two main cell types: stalk cells supporting a spore-containing sorus [1,2]. Spores are protected by a tough cell wall that maximizes survival in adverse conditions. The stalk raises the spore head high enough for the spores to be scattered away to maximize the possibility of germination in a more favorable environment. The dispersion of spores in an environment favorable for successful germination and survival is granted by the ability of the slug to migrate towards light and heat. Other soil micro-organisms such as nematodes could also contribute to the dispersal of spores [3]. The size of the multicellular organism is quite variable, usually ranging from 10,000 to 100,000 cells [4], or up to 2,000,000 [5]; however, slugs composed of as few as 100 cells have been reported [6]. The proportion of spore to stalk cells is usually around 4:1, although the fraction of stalk cells decreases to as low as 10% in larger organisms [7-9]. Formation of the multicellular stages of the Dictyostelids is remarkable in that it is accomplished through the aggregation of neighboring cells rather than by division of a single cell (zygote), as is the case in most multicellular organisms. Its simple life cycle and the mutually exclusive growth and development stages make *Dictyostelium *a popular experimental model to study cellular processes such as chemotaxis, phagocytosis, intra- and inter-cellular communication, cell-cell and cell-substrate adhesion and cellular differentiation. A comprehensive review of the *Dictyostelium *biology has been written in 2001 [10].

With the establishment of a model organism database in 2003, dictyBase [11,12] and the completion of the genome sequence in 2005 [13], *Dictyostelium *research has now entered the post-genomic era. *Dictyostelium *is one of the NIH Model organisms for biomedical research [14] and one of the "reference genomes" selected for comprehensive genome annotation in the Gene Ontology project. In order to improve the consistency of annotations of genes and gene products within dictyBase and across model organism databases, dictyBase curators use biological ontologies. Ontologies can be defined as "collections of formal, machine-processable and human-interpretable representations of entities, and the relations between those entities" [15]. The most well-developed and extensively used ontology is the Gene Ontology (GO) [16,17]. The GO contains over 20,000 terms describing molecular functions, biological processes and cellular components. The objective of the GO is to facilitate the sharing of information across different research communities through a common tool. Other ontologies exist that describe genomic sequences, cells, anatomy, and others entities [18]. Scientific curators review the literature and associate ontology terms to genes and gene products as appropriate.

*Dictyostelium *genes are annotated at dictyBase in a variety of ways, such as the inclusion of Gene Ontology terms and other functional annotations. In this paper, we present an ontological description of the *Dictyostelium *anatomy throughout its life cycle in which we define the structural makeup of *Dictyostelium *and its composing parts, including its different cell types. This ontology allows the annotation of species-specific events, such as phenotypic and gene expression data, that facilitates data retrieval by researchers using tools available at dictyBase. We encourage researchers to use those terms in their publications to provide a uniform vocabulary across the research community. This will ensure accurate annotation and increase the power of the ontology for data mining and analysis.

## Results and Discussion

### The Dictyostelium anatomy ontology

#### High level structure of the ontology

**Figure 1:**
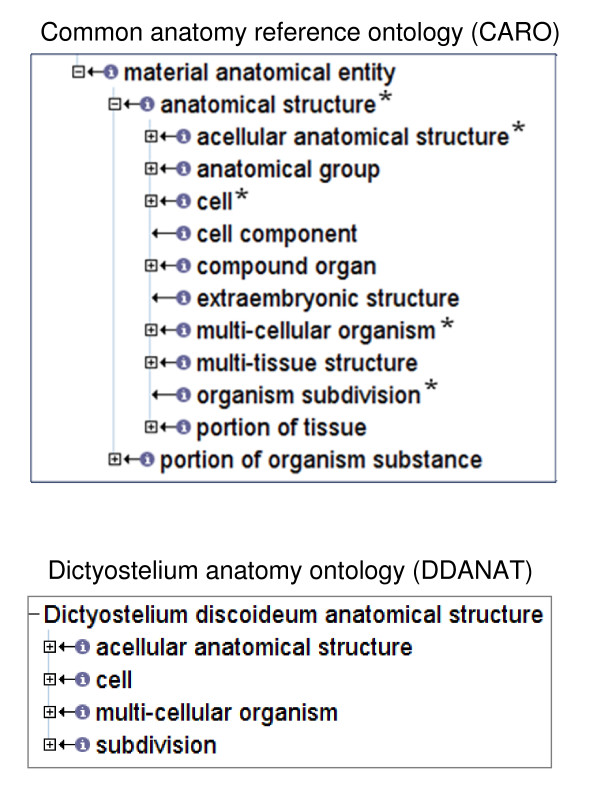
**Comparison of the high level terms of the *Dictyostelium *anatomy ontology and CARO, the Common Anatomy Reference Ontology**. **A**. CARO high level terms. Terms used to make up the *Dictyostelium *anatomy ontology are indicated by stars (*). B. *Dictyostelium *anatomy ontology top nodes.

**Figure 2:**
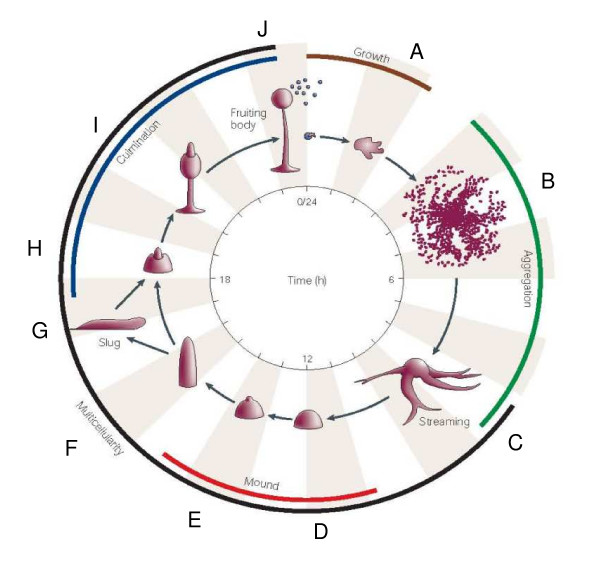
***Dictyostelium *life cycle and the corresponding anatomical structures from the *Dictyostelium *anatomy ontology**. **A**. Vegetative amoebae (DDANAT:0000002). **B**. Aggregation territory (DDANAT:0000003). **C**. Loose aggregate (DDANAT:0000004) with stream (DDANAT:0000013). **D**. Mound (DDANAT:0000005). **E**. Tipped mound (DDANAT:0000006). **F**. Standing slug (DDANAT:0000007). **G**. Migratory slug (DDANAT:0000008). **H**. Early culminant (DDANAT:0000009). **I**. Mid culminant (DDANAT:0000010). **J**. Fruiting body (DDANAT:0000010) with spores (DDANAT:0000414). Modified from [91].

The structure of the *Dictyostelium *anatomy ontology is based upon CARO, the Common Anatomy Reference Ontology, [19] (Figure 1). Four high level terms from CARO were used to construct the *Dictyostelium *anatomy ontology: multi-cellular organism (DDANAT:0010082), subdivision (DDANAT:0010085), cell (DDANAT:0000401), and acellular anatomical structure (DDANAT:0010081). The "multi-cellular organism" branch describes the different developmental stages of the multi-cellular life cycle. Each anatomical structure in the *Dictyostelium *anatomy ontology corresponds to a developmental stage of the multicellular organism or, when appropriate, a part of that structure. The parts of structures, such as "prespore region", additionally have the parent "subdivision". The anatomical structures and subdivisions are themselves composed of either "cells", "acellular anatomical structures", or a mix of both. *Dictyostelium *cells are represented in the Cell Type Ontology [20], also available from OBO, and reciprocally cross-referenced in both ontologies. For example, the vegetative amoebae, DDANAT:0000002 corresponds to CL:0000263 in the Cell Type Ontology. Some cell types exist as unicellular organisms and are thus children of "single cell organism". The *Dictyostelium *anatomy ontology contains 136 terms and 373 relationships. All terms are defined. An overview of the *Dictyostelium *life cycle and the corresponding anatomical structures is shown in Figure 2.

#### The unicellular stage

When nutrients are abundant, a *Dictyostelium *cell exists as a unicellular haploid **vegetative amoeba**, also known as a myxamoeba, (DDANAT:0000002) that feeds on bacteria by phagocytosis and divides by binary fission. Vegetative amoebae are approximately 10–20 microns in diameter and exhibit a rather irregular shape. Unlike their relative *Physarum*, which can form syncitia with multiple nuclei, the vegetative cell is mononucleate. It is defined by a plasma membrane and the absence of a cell wall, and is characterized by the presence of numerous pseudopods and food vacuoles, in concordance with its phagocytic lifestyle [1,21,22]. In the presence of toxic agents, *Dictyostelium *cells undergo major physiological changes, including alteration in the expression of a number of genes as well as morphological changes. Those changes are associated with increased resistance to toxins. These cells are referred to as aspidocytes (DDANAT:0000415) [23].

#### Multi-cellular organism

##### Aggregation

Food depletion triggers physiological changes in the vegetative cell, transforming it into **achemotactic amoeboid cell **(DDANAT:0000402). The chemotactic amoeba produces, secretes and responds to cAMP, the chemoattractant that directs aggregation of amoebae to produce a multicellular tissue [24]. The chemotactic amoeba is highly polarized and moves rapidly in the direction of the chemoattractant [25]. Related organisms either use cAMP or other small molecules such as pterins as chemoattractants [10]. Approximately 4–6 hours after entry into development the **aggregation territory **(DDANAT:0000003), the area covered by a group of chemotactic cells converging toward the same aggregation center, is clearly visible at low magnification. The aggregation territory can reach up to 1 cm in diameter. As they move towards the aggregation center, the cells orient themselves in a head to tail fashion forming **streams **(DDANAT:0000013) and eventually form a loose aggregate [1,26].

The **loose aggregate **(DDANAT:0000004) can be described as the first adherent mass of cells observed during development; it is relatively flat with indistinct borders [1,4]. The molecular mechanisms controlling aggregation have been recently reviewed [27,28] and many of the terms reflecting those events are present in the Gene Ontology such as cAMP-mediated signaling (GO:0019933) and chemotaxis to cAMP (GO:0043327). Most of the cells making up the loose aggregate are undifferentiated and are referred to as **aggregate cells **(DDANAT:0000403). Cell-type specific gene markers start being expressed in a minority of cells at the loose aggregate stage: **prespore cells **(DDANAT:0000405), **pstA cells **(DDANAT:0000408), **pstO cells **(DDANAT:0000407), expressing the ecmA gene from the proximal and distal part of the promoter, respectively), as well as cells expressing the *ecmB *gene, **pstB cells **(DDANAT:0000417). Many of the pstA cells appear to be transiently located at the periphery of the loose aggregate while the other prestalk cells appear to be distributed randomly [29-32]. The **surface sheath of the loose aggregate **(DDANAT: 0000014) is formed late during aggregation. It has a slimy appearance and is composed of polysaccharides and proteins [22,33,34]. The surface sheath appears to hold the cell mass together thus helping to shape the next stage: the hemispherical tight aggregate or **mound **(DDANAT:0000005). The mound stage is characterized by cellular movements that result in the formation of distinct subdivisions or zones within the organism: the prestalk and the prespore zones. It is believed that positive chemotaxis to cAMP signals originating from the **apex of the mound **(DDANAT:0000109) cause pstA and pstO cells to move to the top of the mound. From there they form themselves into a nipple-like tip, the **tip-organizer of the tipped mound **(DDANAT:0000080) giving rise to the **tipped mound **(DDANAT:0000006).

##### Prestalk and prespore zones

**Figure 3:**
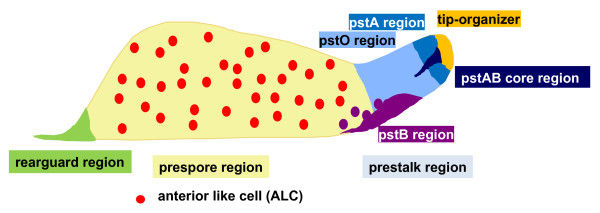
**Subdivisions of the multicellular organism**. The prestalk and prespore zones are recognizable from the tipped mound stage. This diagram represents the different subdivisions of the multicellular organism at the migratory slug stage. The subdivisions remain in the same relative positions and proportions until culmination.

The prestalk and prespore zones become clearly defined by the tipped mound stage and remain in the same relative positions and proportions until culmination as shown in Figure 3 using the migratory slug as an example. The **prestalk region **(DDANAT:0000087) is located at the apical-most part of the organism in the tipped mound and in the standing slug, and at the anterior region in the migratory slug. It contains **prestalk cell**s (DDANAT:0000406), undergoing differentiation into stalk cells. The prestalk region can be subdivided into four zones: the uppermost **prestalk A region **(DDANAT:0000088); the **prestalk O **region (DDANAT:0000092), located just underneath the prestalk A zone, the **prestalk AB core region **(DDANAT:0000091), a cone-shaped area located at the core of the prestalk A zone, and the **tip-organizer**. Cells within those regions are referred to as pstA, pstO and pstAB cells, based on the markers they express: **pstA **(DDANAT:0000408) and **pstO **(DDANAT:0000407) cells respectively express ecmA from the proximal and distal part of its promoter, while **pstAB **(DDANAT:0000410) cells express both *ecmA *and *ecmB *[29-31,35,36]; note that the expression data from the In Situ Hybridization Atlas [36] can be viewed at dictyBase from the Gene Page of every gene used for that study. There is also a part of the prestalk zone that is defined functionally based on its ability to control the behavior of the multicellular organism and that we will term the **tip-organizer **(DDANAT:0000103) The tip-organizer ensures the morphological integrity of the organism; it suppresses the formation of other tips and if a second tip is grafted onto a slug flank it will direct the formation of a secondary slug [4,37-39]. At the migratory slug stage the tip-organizer is necessary for locomotion of the organism and it contains the light sensitive areas that direct slug phototaxis [40]. The tip-organizer is also the region that determines the timing of entry into culmination [41]. Based on the expression pattern of the *cudA *gene, the tip-organizer region forms a cone that extends right up to the most anterior part of the slug tip [42,43]. The **prespore region **(DDANAT:0000086) is located basally relative to the prestalk region (posteriorly in the migratory slug stage) and is primarily composed of **prespore cell**s (DDANAT:0000405). The prespore region also contains cells that have some of the properties of prestalk cells (located at the anterior, prestalk region of the slug) and are thus called **anterior-like cell**s (DDANAT:0000404). Cell fate is not determined until late in development and expression of these markers cannot be used to predict what cell type a cell will differentiate into: at the slug stage, they may localize to the anterior region and become pstA cells, pstO cells or pstAB cells, move to the basal region (rearguard cells) or they may remain scattered in the prespore zone as anterior-like cells [29-31,44].

##### Slug stage

Upon completion of the aggregation stage, the cell mass rises upwards forming a structure called the **standing slug**, the first finger or, more simply, the finger (DDANAT:0000007). The **prestalk region of the standing slug **(DDANAT:0000019), derives directly from the prestalk region of the tipped mound. It is composed of the four regions described above, **tip-organizer**, **prestalk A**, **prestalk O**, and **prestalk AB core region of the standing slug **(DDANAT:0000020-23). Below the prestalk region lies the **prespore region of the standing slug **(DDANAT:0000024) which makes up the bulk of the cell mass and is composed mostly of prespore cells and of a small proportion of anterior-like cells [45,46]. The **basal region of the standing slug **(DDANAT: 0000025) is composed of pstB cells that express *ecmB *and show very little *ecmA *expression. The pstB cells remain at the base if the standing slug enters directly into culmination where they form part of the outer basal disc. [32,47].

The *Dictyostelium *slug has the ability to migrate if the conditions are not optimal for the completion of development, forming a **migratory slug **(DDANAT:0000008), also knows as a pseudoplasmodium or simply, slug. The length of this stage is variable and it is sometimes absent [4,48]. The transition from the standing slug to the migratory slug occurs as the standing slug bends from a vertical position to a horizontal position. The slug is cylindrical in shape and under standard laboratory conditions usually measures between 0.8 to 1.2 mm in length with a diameter of 0.15 to 0.25 mm. Slugs exhibit positive taxis towards light and heat, and do not enter culmination until the conditions are optimal. The slug can move at a speed of 0.5 to 2 mm per hour. It has a rounded, tapering region at its anterior end that is raised with respect to the substratum and that contains the **prestalk region of the migratory slug **(DDANAT:0000028) [1,4]. The **prestalk AB core region of the migratory slug **(DDANAT:0000032) is, however, variably present because it is periodically shed from the back of the slug [49]. It is probably replaced by further differentiation of pstA cells, which start to express *ecmB*, demonstrating the highly dynamic nature of cell differentiation in *Dictyostelium*. For an illustration of the migratory slug see Figure 3.

The **prespore region of the migratory slug **(DDANAT:0000033) makes up the posterior three quarters of the organism; in addition to prespore cells, the prespore region also contains anterior like cells [46]. The very **posterior region of the migratory slug **(DDANAT:0000034), also known as the rearguard region is variable from organism to organism. When observed, it contains a subtype of pstB-expressing anterior-like cells, **rearguard cells **(DDANAT:000409), that express *ecmB *and eventually contribute to the outer basal disc. The presence of rearguard region is variable when the slug elects to migrate away from its origin, being left on the substratum with the slime trail. A new population of pstB cells appear, clustered at the boundary between the prespore and the prestalk regions and with a highly dynamic anterior-posterior movement pattern that leads to the replacement of the rearguard region [32,47].

The slug is surrounded by a **peripheral layer of the migratory slug **forming a coherent tissue (DDANAT:0000035) composed of electron-dense cells that are connected to each other, the **peripheral layer cell**s (DDANAT:0000095). Their shape varies depending on their location within the organism, taking the shape of the underlying areas: the cells in the prestalk zone have a basal-apical polarity and contain irregular inward projections, while cells in the prespore zone are flat and elongated [50]. No molecular markers have yet been identified for this cell type.

The **surface sheath of the migratory slug **(DDANAT:0000036) is a protective layer of cellulose (60%), protein (15%), and polysaccharides about 10–50 nm thick. Compared to that of the aggregate, the surface sheath of the slug has a higher protein content and contains certain sugars, including galactose, fucose and *N*-acetylglucosamine that are not found in aggregates [34,51]. The surface sheath is thinnest at the tip and increases in thickness as the distance from the tip increases. The surface sheath holds the cell mass together and possibly provides adhesion to the substratum to allow migration. Fragments of the surface sheath are left behind the slug during migration producing a **slime trail **(DDANAT:0000076) that can be up to 2–3 cm in length. The surface sheath is continually being synthesized to replace the fragments left behind [1,33,52-55].

##### Culmination

**Figure 4:**
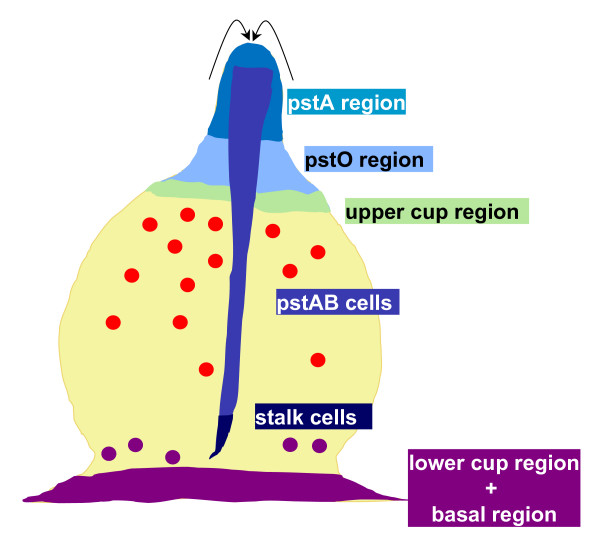
**Cell movements during culmination**. Terminal cell differentiation takes place during the culmination stage and is correlated with cellular movements within the organism, as shown here for an early culminant. PstAB cells present in the slug are the first to migrate down the stalk tube and terminally differentiate into stalk cells, hence referred to as primary prestalk cells. They are replaced by pstA cells, which start expressing *ecmB *and in turn become stalk cells (secondary prestalk cells). PstA cells are in turn replaced by pstO cells. This process continues until all prestalk cells have been incorporated into the stalk.

The final stage of development is called culmination and it is characterized by a number of morphological changes that result in the formation of the fruiting body, sometimes called the sorocarp. In the Gene Ontology, the Dictyostelid fruiting body is called the sorocarp to distinguish it from bacterial or fungal fruiting bodies. The fruiting body consists of a stalk (sorophore) supporting a mass of spores [1]. Culmination can be broken down into three distinct morphological stages: early culmination, mid culmination, and late culmination. Main cellular movements that occur during culmination are shown in Figure 4.

###### Stalk formation

One of the major events of culmination is the formation of the **stalk **(DDANAT:0000093), a cylinder of dead, vacuolated stalk cells surrounded by a proteo-cellulose casing: the **stalk tube **(DDANAT:0000097). The stalk composition is similar to that of the surface sheath: approximately 50% cellulose, 15% protein and 35% polysaccharides and fatty acids [56]. Cells within the stalk tube, the **stalk cell**s (DDANAT:0000413), are around 8 microns in diameter, polyhedric, highly vacuolated, and are surrounded by a cell wall composed of cellulose. Stalk cells provide structural support for the fruiting body and die by autophagic cell death during their terminal differentiation [57].

###### Early culmination

Culmination begins when the standing slug elects for immediate culmination or when the migratory slug ceases movement. In the latter case the movement arrest starts at the anterior region, such that cells from the posterior region, that are still moving forward, join the anterior portion of the organism, forming the **early culminant **(DDANAT:0000009). The cell mass flattens downward and in so doing, comes to resemble a Mexican hat. The **prestalk region of the early culminant**, (DDANAT:0000037), which in the migratory slug is at a 0–45° angle relative to the substratum, therefore rotates to become perpendicular with respect to the substratum [1]. Formation of the stalk begins with the formation of the stalk tube during early culmination. The **stalk tube of the early culminant **(DDANAT:0000043) first appears as a thin, translucent membrane at the center of the cell mass. The stalk tube is deposited extracellularly, most likely by both the pstA cells and pstO cells [4,22,29,49,57-60]. The ALC/pstB cells (anterior like cells expressing *ecmB*), scattered in the **prespore region of the early culminant **(DDANAT:0000041), migrate down to form the **lower cup region of the early culminant **(DDANAT:0000113), just above the **basal region of the early culminant **(DDANAT:0000045). A discrete sub-set of ALC/pstB that express *ecmB *from a distal element of its promoter migrate upwards to form the **upper cup region of the early culminant **(DDANAT:0000042), hence becoming **upper cup cell**s (DDANAT:0000112) [61,62].

###### Mid-culmination

Important morphological changes take place in the **mid culminant **(DDANAT:0000010): the stalk tube extends downwards, while the nascent spore mass begins its ascent up the stalk with the upper cup cells acting as a form of "motor" [63]. The formation of the **stalk of the mid culminant **(DDANAT:0000052) continues through the differentiation and the deposition of stalk cells on top of one other within the **stalk tube of the mid culminant **(DDANAT:0000053). Progression towards terminal stalk cell differentiation occurs sequentially: the pstAB cells in the core region of the tip move down through the culminant into the stalk tube (Figure 4). They produce increasing amounts of cellulose and become terminally differentiated into stalk cells. As the first cells to reach the substratum, they eventually form the inner basal disc. The major prestalk differentiation process occurs as pstA and pstO cells migrate in a "reverse fountain" movement: the **prestalk O region of the mid culminant **(DDANAT:0000050) which forms a ring just beneath the prestalk A region, contains pstO cells that migrate upwards towards the tip, differentiating into pstA cells, then into pstAB cells, and finally terminally differentiate into stalk cells. Cells from the **prestalk A region of the mid culminant **(DDANAT:0000049), located at the apex of the organism, differentiate into pstAB cells as they migrate downwards into the entrance of the stalk tube. As they progress down the stalk tube, the pstAB tube cells produce increasing amounts of cellulose and become terminally differentiated into stalk cells. The continued layering of stalk cells on top of each other results in the upward extension of the stalk.

As the **prespore region of the mid culminant **(DDANAT:0000054) rises to form the sorus, it appears to separate from the supporting base, giving the latter a disc-like appearance known as the **basal disc of the mid culminant**, DDANAT:0000057. Using appropriate prestalk-specific markers, the formation of the upper and lower cups is already visible, as ALC/pstB anterior-like cells localize to the **upper cup region of the mid culminant **(DDANAT:0000055) and the **lower cup **region of the mid culminant (DDANAT:0000056) [64].

###### Late culmination

The **late culminant **(DDANAT:0000011) is the stage at which the sorogen (spore head) ascends the stalk and terminal differentiation of spores occurs, while prestalk cells continue to differentiate into stalk cells [1,2,57]. Prespore cells move to the top of the stalk [63,65] and, terminally differentiate into spores. At this stage, the spores are contained in a more compact structure, the **sorus of the late culminant **(DDANAT:0000061). Differentiated spores have a characteristic elliptical shape and are approximately 6–9 microns by 3 microns. They are protected by a tri-layered spore coat, produced from the prespore vesicles of the prespore cells, contained within the slug. Prespore vesicles fuse with the plasma membrane during culmination and the proteins present in the vesicles are secreted to form the spore coat. The inner and the outer layers contain glycoproteins as well as galactose/N-acetylgalactosamine polysaccharide (GPS), while the middle layer is composed of cellulose [1,58,66,67]. The sorus is bounded above by the **upper cup **(DDANAT:0000063) and below by the **lower cup of the late culminant **(DDANAT:0000064) [46,62]. Towards the end of culmination many of the upper cup cells enter the stalk tube and become part of the upper stalk but some remain excluded from the tube, forming a button of cells atop the stalk that persists in the mature organism. This is the **apical disc of the late culminant **(DDANAT:0000062) [68]. The organism is supported by the **basal disc of the late culminant **(DDANAT:000065), a cone-shaped cell mass that anchors the organism to the substratum. The **stalk of the late culminant **(DDANAT:000059) extends as long as prestalk cells are available until it reaches and embeds itself into the basal disc, forming the **inner basal disc of the late culminant **(DDANAT:0000066). It is surrounded by the **outer basal disc **(DDANAT:0000067) [32,49].

##### Mature fruiting body

Completion of development in *Dictyostelium *gives rise to the mature **fruiting body **(DDANAT:0000012) that usually measures between 1.5 and 3 mm in height. The lemon-shaped **sorus of the fruiting body **(DDANAT:0000070), the spore-bearing structure, sits atop the stalk. The sorus is about 125–300 microns in diameter and pale yellow in color. By the time the fruiting body is mature the slime sheath that surrounds the organism earlier in development disappears and spores are held together solely by surface tension [69]. The **apical disc **and the **lower cup of the fruiting body **(DDANAT:0000071 and DDANAT:0000072, respectively) arise from corresponding structures of the late culminant. The upper cup is no longer present as it has completely been absorbed into the stalk. In the mature fruiting body all the prestalk cells have entered the stalk, making it longer and thinner than in immature organisms. The **stalk of the fruiting body **(DDANAT:0000068) is enclosed in the **stalk tube **(DDANAT:0000069) and is usually 5–15 microns in diameter and between 1.5 to 3 cm in height. The **basal disc of the fruiting body **(DDANAT:0000073) is flatter and wider than that of the late culminant: it is around 150 to 400 microns in diameter and is covered by the **surface sheath of the fruiting body **(DDANAT:0000079) [57,69].

##### Completion of the life cycle

**Spore**s (DDANAT:0000414) have very low metabolic activity and can survive several years without nutrients. The spores remain in a dormant state as long as environmental conditions remain unfavorable. Once dispersed into the environment and given proper environmental conditions, germination takes place. The phase during which spores prepare to germinate is called activation. The first visible event in germination is spore swelling, which is followed by the emergence of the amoebae from the spore coat. The process takes between two and six hours. The freed amoebae reenter the vegetative stage of the life cycle, thus completing the life cycle [1,70,71].

### Sexual cycle

Under conditions of high humidity and darkness, *Dictyostelium *cells can undergo a sexual cycle during which two cells of opposite mating types fuse, forming a **giant cell **(DDANAT:0000121). The giant cell attracts neighboring cells by cAMP signaling and engulfs them by phagocytosis thus becoming a large multinucleate cell, ranging in diameter from 35 to 90 microns. During the maturation of the giant cell, the engulfed cells (endocysts) are digested and nuclear fusion occurs to form a true zygote, the **macrocyst **(DDANAT:0000084) [72-77]. The macrocyst is protected by a three-layer coat: the inner layer is deposited by the aggregating cell, while the other two are deposited by the zygote after phagocytosis. All three layers are composed of cellulose and different sets of glycoproteins. In addition, the outer spore coat contains a galactose/N-acetylgalactosamine polysaccharide (GPS) [67]. Germination of the macrocyst releases diploid amoebae that later divide by meiosis to give rise to a vegetative amoeba [78].

### Annotating phenotypes using controlled vocabularies

The value of the *Dictyostelium *anatomy ontology is its use to annotate the phenotypes of mutant strains at dictyBase. We have adopted the 'Entity-Quality' model to construct formal phrases that describe mutant phenotypes of *Dictyostelium *strains. This model uses two controlled vocabularies to describe the defects in functions, processes, or anatomical structures of mutants: (1) a vocabulary describing the entity being observed in the mutant, and (2) a vocabulary qualitatively describing the defect of the mutant. The qualities can be, for example: abolished, decreased size, increased rate [79]. The ontologies describing Entity can be taken from the Gene Ontology, to describe general biological processes such as cytokinesis and phagocytosis. Other terms can be taken from ChEBI (Chemical Entities of Biological Interest), describing small molecules such as cAMP and myo-inositol monophosphate [80]. The advantage of this approach is that consistent use of well-defined terms allows bulk querying of specific phenotypes or types of defects. We use the *Dictyostelium *anatomy ontology to describe developmental events or *Dictyostelium*-specific events, such as aggregation defects (abolished aggregation, delayed aggregation, precocious aggregation), aberrant cell type differentiation (abolished, increased, decreased), etc.

**Figure 5:**
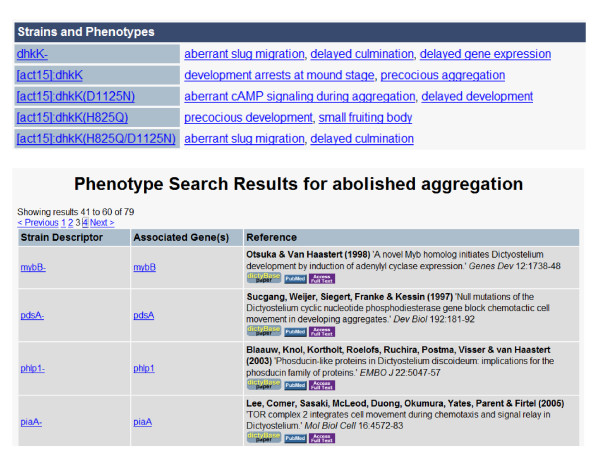
**Phenotype annotations in dictyBase**. All phenotypes are constructed using the Entity-Quality model. A. Each dictyBase Gene Page has a section listing strains and phenotypes relevant to that gene. B. Querying dictyBase for phrases such as 'abolished aggregation' returns all strains annotated to that term, the gene(s) mutated in that strain, and the reference describing the phenotype. Show here are four annotations to 'abolished aggregation' out of 79 strains annotated to that phenotype.

An example of how data from a research article is converted to annotations is shown in Figure 5. A study of the histidine kinase DhkK shows that dhkK-, a knock out mutant exhibits a delay in culmination, while overexpression of the same gene product from a constitutively active promoter, [act15]:dhkK accelerates culmination. The importance of the residues involved in signal transduction was also studied: the histidine phospho-acceptor site (H825Q), as well as the phospho-acceptor site (D1125N) were mutated and overexpressed. The H825Q mutation ([act15]:dhkK(H825Q)) accelerated culmination, while mutation of the phospho-acceptor site delayed culmination in a dominant fashion, as it was also observed in the double mutant ([act15]:dhkK(H825Q/D1125N)).

### Using the ontology

Users may download the ontology [81], browse it using the EBI Ontology Lookup Service [82,83], or view all terms at dictyBase [84]. Currently, the *Dictyostelium *Anatomy Ontology is used for annotation of phenotypes at dictyBase. As of January 2008, there are 1075 strains corresponding to 567 genes that have phenotype annotations in dictyBase. We have a total of 2771 phenotypic observations for those 1075 strains. There are several ways to view annotations at dictyBase. Strains are listed on the relevant dictyBase Gene Page together with their phenotypic annotations. It is also possible to search on strains directly; for example, searching for 'dhkK' results in one gene and 14 strains. In addition, the ontology terms may be searched using the dictyBase general search box. For example, searching for "aggregation" leads to all different phenotypic defects related to aggregation: aberrant aggregation, delayed aggregation, precocious aggregation, abolished chemotaxis to cAMP during aggregation, aberrant regulation of aggregate size, etc. Figure 5B shows a number of mutant strains annotated to "abolished aggregation". The complete list can be obtained by searching for "abolished aggregation" in the dictyBase search box. In addition to searching for specific processes, the Entity-Quality model also allows queries for types of defects; for example, one can look for mutants showing different delays in development (*delayed *aggregation, *delayed *culmination, *delayed *tip formation). Lists of all terms used for phenotypic annotations and annotations of mutant strains can be viewed at the dictyBase Downloads site [85]. The anatomy and phenotype terms should be useful to *Dictyostelium *biologists as they describe developmental processes in their publications. To maximize computational modeling of *Dictyostelium *development we encourage biologists to use this ontology as they describe mutants.

### Future directions: Integrating GO processes in the Dictyostelium anatomy ontology

The *Dictyostelium *anatomy ontology presented here describes the different parts composing the *Dictyostelium *organism at its different developmental stages. The next step in the representation of the *Dictyostelium *life cycle is to add links to the GO biological processes involved at each stage of the life cycle. For instance, the aggregation stage is mediated by complex signaling events: the production and secretion of cAMP that binds to specific membrane receptors and activates G-proteins. Numerous other factors are activated that eventually result in cellular movement towards the cAMP source, leading to the formation of aggregate. We have already been using GO process and function terms for phenotype annotation, for example "decreased chemotaxis to cAMP", "aberrant cAMP-mediated signaling", "decreased F-actin polymerization", etc. Adding the links between the two ontologies will ensure that both ontologies remain synchronized.

## Methods

The *Dictyostelium *anatomy ontology was developed using OBO-Edit [86]. Obol [87] was used to check for correct parentage of combinatorial terms. The ontology applies the principles of the Open Biomedical Ontologies (OBO) Foundry [88]. Specifically: the ontology is publicly available; it uses the OBO syntax; it has unique identifiers; terms are defined and related to each other using OBO relations. Three relationship types are used: is_a, part_of and develops_from [89]. Each term has an is_a relationship with one of the root terms. For example, every developmental structure has is_a relationships with the term "multi-cellular organism" (DDANAT:00100082): the aggregation territory (DDANAT:0000003) and the fruiting body (DDANAT:00100012) are types of *Dictyostelium *"multi-cellular organism". The components of the different anatomical structures, such as prespore and prestalk regions of the migratory slug (DDANAT:0000033 and DDANAT:0000028, respectively), have a minimum of two relationships: is_a "Dictyostelium discoideum subdivision" (DDANAT:0010085), as well as being 'part_of' the migratory slug (DDANAT:0000008). Also, each developmental stage has a 'develops_from' relationship with respect to the immediately preceding stage or structure: for instance, the fruiting body (DDANAT:0010012) develops from the late culminant (DDANAT:0010011) and the sorus of the fruiting body (DDANAT:0000070) develops_from the sorus of the late culminant (DDANAT:0010061). The ontology is available for download in OBO and OWL format at the Open Biomedical Ontologies (OBO) Foundry site [81]. As knowledge evolves, the ontology will be updated. Researchers can submit suggestions for modifications and updates to the ontology using the Dicty anatomy Source Forge tracker [90].

## Authors' contributions

PG and JGW developed the ontology and wrote the manuscript. PG converted the ontology in OBO edit format. PF and RLC helped with ontology development and in writing the manuscript. All authors read and approved the final manuscript.
